# Influence of Nutritional Parameters on the Evolution, Severity and Prognosis of Critically Ill Patients with COVID-19

**DOI:** 10.3390/nu14245363

**Published:** 2022-12-16

**Authors:** Yenifer Gamarra-Morales, Jorge Molina-López, Juan Francisco Machado-Casas, Lourdes Herrera-Quintana, Héctor Vázquez-Lorente, José Castaño-Pérez, José Miguel Perez-Villares, Elena Planells

**Affiliations:** 1 Clinical Analysis Unit, Valle de los Pedroches Hospital, Pozoblanco, 14400 Córdoba, Spain; 2 Faculty of Education, Psychology and Sports Sciences, University of Huelva, 21007 Huelva, Spain; 3 Intensive Care Unit, Virgen de las Nieves Hospital, Fuerzas Armadas Avenue, 18014 Granada, Spain; 4 Department of Physiology, Faculty of Pharmacy, Institute of Nutrition and Food Technology “José Mataix”, University of Granada, 18071 Granada, Spain

**Keywords:** COVID-19, nutritional status, nutritional biomarkers, morbidity, mortality

## Abstract

This study evaluated the clinical and nutritional status, the evolution over three days, and the relationship between nutritional, inflammatory, and clinical parameters of critically ill patients with COVID-19. A longitudinal study was conducted in the Intensive Care Unit of the Virgen de las Nieves University Hospital in Granada (Spain). The study population comprised patients with a positive polymerase chain reaction test for COVID-19 presenting critical clinical involvement. Clinical outcomes were collected, and inflammatory and nutritional parameters (albumin, prealbumin, transferrin, transferrin saturation index, cholesterol, triglycerides and Controlling Nutritional Status (CONUT) score) were determined. A total of 202 critical patients with COVID-19 were selected, presenting highly altered clinical-nutritional parameters. The evolution experienced by the patients on the third day of admission was a decrease in albumin (*p* < 0.001) and an increase in prealbumin (*p* < 0.001), transferrin (*p* < 0.002), transferrin saturation index (*p* < 0.018), and cholesterol (*p* < 0.001). Low levels of albumin, prealbumin (on the third day) and high CONUT score (on the third day) showed an association with higher mortality. Nutritional variables were inversely correlated with clinical and inflammatory parameters. Critically ill patients with COVID-19 have poor nutritional status related to a poor prognosis of disease severity and mortality.

## 1. Introduction

The novel coronavirus disease 2019 (COVID-19) is the condition responsible for the global pandemic caused by severe acute respiratory syndrome coronavirus 2 (SARS-CoV-2). The COVID-19 pandemic has caused an enormous health and economic impact and changed how medicine and medical education are practiced. This transformation is based on the following reflections on the negative impact that the COVID-19 pandemic has had on medical practice [1]: (1) the publication of false and sensational news [2]; (2) the risks of non-evidence-based medical decision-making [3]; (3) the bioethical implications when there are not enough resources to go around [4,5]; and (4) The possible effects of the crisis on the teaching of medicine. The symptoms manifested by patients with COVID-19 can negatively impact the nutritional status and prognosis of patients [6,7]. The severity of COVID-19 is positively associated with excessive systemic inflammation and immune response and, consequently, with oxidative stress, coagulation disorders, and multi-organ failure [8,9,10]. In addition, malnutrition has been one of the most common complications of older survivors of COVID-19 [11]. Some publications mention the importance of investigating specific aspects of nutrition in patients with COVID-19 admitted to the Intensive Care Unit (ICU) [12,13].

Malnutrition in hospitalized patients remains a relevant problem in Spain due to its implication in clinical worsening during disease evolution, especially in critical patients, affecting 30–50% [14]. Various studies [15,16,17,18,19,20] show that malnutrition is currently underestimated and undertreated in the general hospital population. A timely diagnosis and an early identification of malnutrition are essential in hospitalized patients to reduce complications, especially infections, length of hospital stay, and mortality, thus helping to improve the clinical course by providing adequate nutritional support [21,22]. The nutritional assessment can be performed by examining some analytical parameters that will determine the nutritional status of the patient before and during admission to the ICU. Performing this assessment is very difficult because the parameters used to assess the nutritional status are influenced by the support treatment and the metabolic alteration suffered by these critical patients [23]. Albumin and prealbumin should not be used individually to assess the nutritional status, and they are also indicative parameters of the inflammatory state. However, their levels are easily measured in clinical practice, and their determination can guide clinicians regarding the nutritional status and evolution of the patient in conjunction with other clinical and analytical parameters.

Low albumin levels are related to a negative prognosis, with an increase in the appearance of complications and mortality. However, albumin levels have little sensitivity to changes in nutritional status, so it is not a suitable parameter for short-term nutritional monitoring. Values below 2.1 g/dL indicate a serious malnutrition situation [23]. Prealbumin plasma levels decrease in malnutrition, infection, and liver failure. Prealbumin has a short half-life (2 days); this makes it a good parameter for evolution and follow-up in critically ill patients [24]. Transferrin levels are not useful for evaluating the nutritional status, but their values are decreased in liver disease, sepsis, and intestinal disease. Lipids, total cholesterol, low-density lipoprotein (LDL), high-density lipoprotein (HDL), triglycerides, Apo A1, Apo B, lipoprotein lipase A, and the fatty acid profile can be measured in plasma, but they are not parameters for evaluating the nutritional status. In malnourished patients with renal and hepatic insufficiency and malabsorption syndrome, mortality is increased with high cholesterol levels.

Therefore, the objective of the present study was to investigate the usefulness of some nutritional parameters in the evolution and prognosis of critically ill patients with COVID-19 admitted to the ICU. In addition, we aimed to determine the evolution of nutritional parameters such as albumin, prealbumin, transferrin, transferrin saturation index (TSI), cholesterol, and triglycerides on the third day of admission to the ICU. We also aimed to investigate their usefulness as potential biomarkers of mortality and disease severity.

## 2. Materials and Methods

### 2.1. Study Design and Participants

The study design is based on a prospective observational follow-up study of critically ill patients with COVID-19. Patients admitted to the ICU with a diagnosis of COVID-19 from March to December 2020 were recruited for this study. The diagnosis of the patients was based on a positive polymerase chain reaction (PCR) test and subsequent RNA sequencing specific for the SARS-CoV-2. The patients were selected at the Hospital Virgen de las Nieves ICU in Granada (Spain). This study was conducted in accordance with the Declaration of Helsinki and approved by the Ethics Committee of the hospital (Ref. number: 149/TEIH/2016). Informed consent was obtained from the patients or their relatives.

Patients over 18 years of age who were admitted to the ICU, stayed for at least 3 days and presented a positive PCR test forSARS-CoV-2 were recruited. Those patients under 18 years, those who were pregnant, and those who did not have a positive PCR test even though they presented symptoms compatible with COVID-19 were excluded. Patients were considered critically ill when they had a respiratory failure requiring mechanical ventilation, needed vasopressor treatment (shock), or presented other complications with organ failure requiring monitoring or treatment in the ICU.

### 2.2. Data Collection

Basic information on the study patients (age, sex, and comorbidities) and their illness and evolution in the ICU (date of onset and discharge from the ICU, PCR results against COVID-19) was collected. The clinical parameters collected on the first and third day of admission to the ICU were obtained in the same way. The clinical parameters collected were: Acute Physiology and Chronic Health Evaluation II (APACHE II) scale score [25], Sequential Organ Failure Assessment (SOFA) scale score [26], Controlling Nutritional Status (CONUT) score [27], days of mechanical ventilation, days of ICU stay, and mortality at 28 days. Heart rate, blood pressure, respiratory rate, and other respiratory function variables such as FiO_2_ and PaO_2_/FiO_2_ were also obtained. One of the limitations of our study is that we have not been able to calculate other nutritional risk indices due to the lack of data on the patient’s body mass.

Serum samples were obtained from patients included in the study on admission to the ICU and on the third day. The samples were centrifuged, and after obtaining the serum, the parameters were determined immediately. The laboratory variables analyzed were (a) biochemical variables: sodium, potassium, creatinine, total proteins, lactate dehydrogenase (LDH), alanine aminotransferase (ALT), aspartate aminotransferase (AST), gamma-glutamyl transferase (GGT), alkaline phosphatase, creatine kinase (CK); (b) hematological variables: hemoglobin, hematocrit, leukocytes, percentage of neutrophils, percentage of lymphocytes, platelets, International Normalized Ratio (INR) and activated partial thromboplastin time (APTT); (c) nutritional variables: glucose, total proteins, albumin, prealbumin, transferrin, TSI, cholesterol, and triglycerides; (d) other variables such as inflammatory markers: fibrinogen, D-dimer, C-reactive protein (CRP), ferritin, and procalcitonin (PCT). These parameters were analyzed using the Abbott Core Laboratory^®^ autoanalyzer for biochemistry and immunochemistry” Alinity.” The determination methods were enzymatic colorimetry and immunoassay.

### 2.3. Treatment and Nutritional Support

The patients received treatment that included medications (antivirals, antibacterials, corticosteroids, etc.), respiratory support, and nutritional support (parenteral, enteral, or both) during their hospital stay. The latter was according to the Clinical Nutrition Units guidelines of the hospitals, based on the American Society for Parenteral and Enteral Nutrition (ASPEN) and the European Society of Parenteral and Enteral Nutrition (ESPEN) guidelines [28]. Parenteral nutrition consisted of administering at least 2 energy-providing nutrients, including glucose, fat emulsion, and amino acids, for at least 3 days, providing > b10 kcal/kg/d of energy. The enteral nutrition provided to the study patients consisted of commercial formulas fed orally or tube fed for at least 3 days, providing > b10 kcal/kg/d of energy. Caloric administration during the early phase was hypocaloric, without exceeding 70% of energy expenditure as recommended by the ESPEN [6].

### 2.4. Statistical Analysis

A database was completed, and the statistical tests were processed using the statistical program SPSS version 21.0. The sample size calculation was performed to evaluate the difference between two means for a paired t-test, an alpha of 0.05, a power of 95% and a medium effect size (0.05). A total of 45 participants were required. However, a total of 202 participants were recruited for the present study; since it is a cross-sectional study, it does not involve any additional intervention for the patient, and therefore, ethical aspects are not compromised. Qualitative variables were presented as frequencies and percentages, and quantitative variables as mean ± standard deviation (SD) or median (Interquartile range). The assumption of normality was tested using the Kolmogorov–Smirnoff–Lilliefors test. The variables treated as non-parametric in this study were alanine aminotransferase, aspartate aminotransferase, glutamyl transferase, lactate dehydrogenase, creatine kinase, and days of mechanical ventilation; these variables were presented as median (interquartile range). The relationship of the dichotomous qualitative variables was found using the Chi-square test. The association of quantitative variables with mortality and mechanical ventilation was performed using the Student’s t-test. Quantitative variables between day 1 and day 3 of admission were compared using the Student’s t-test for paired data to check the evolution of the critically ill patients with COVID-19 in the ICU, and the Wilcoxon test was used for non-parametric variables. Correlations between nutritional parameters and clinical variables were established using Pearson’s correlation coefficient for parametric variables and Spearman’s correlation coefficient for non-parametric variables. A *p*-value < 0.05 was considered statistically significant.

## 3. Results

A total of 202 patients requiring admission to the ICU, staying there for at least 3 days, and meeting all the inclusion criteria were recruited. The study sample comprised 147 (72.8%) men and 55 (27.2%) women. Significant differences were observed in the frequency of men and women with this disease admitted to the ICU, being more frequent in men (chi-square = 22.1; *p* < 0.001). The mean age (SD) was 60.6 (13.6) years. Most patients diagnosed with COVID-19 presented dry cough, fever, asthenia, myalgia, ageusia, and anosmia. Admission to the ICU was due to respiratory failure in 98% of cases. Most patients had one or more underlying diseases. Among patients with comorbidities, 35.1% had diabetes, 54.1% had hypertension, 29.7% had dyslipidemia, 5.4%had chronic kidney disease, 27.0% had chronic obstructive pulmonary disease, and 16.2%had cardiovascular disease.

The mean (SD) length of stay in the ICU was 21.6 (16.6) days and the mean (SD) number of days on mechanical ventilation was 17.3 (16.2) days. A total of 156 (77.2%) patients required mechanical ventilation (these patients received vasoactive support), and 44 (21.8%) patients required a high-flow nasal cannula (HFNC). Therefore, a total of 200 (99.0%) patients required ventilatory support. The kidney also received support in our patients; 10 (5.0%) patients required extrarenal purification techniques. A total of 5 (2.5%) patients required parenteral nutrition, whereas 151 (74.8%) patients required enteral nutrition.

In the first 59 cases (until May 2020), all patients received the standard treatment recommended without scientific evidence (antivirals and antibiotics). However, because of the publication of the RECOVERY study in July 2020 [29], none of the patients received antivirals, and only 63 (31.2%) patients received antibiotics. In addition, all these patients but two received dexamethasone at the RECOVERY study dose because all but two were admitted due to respiratory failure. The RECOVERY study showed that 6 mg/day of dexamethasone decreases mortality in COVID-19 patients requiring oxygen therapy. In contrast, mortality was higher if dexamethasone was prescribed in those who did not need oxygen therapy.

Table 1 shows the clinical characteristics on the first and third days, and the fourth column shows the evolution after 3 days in the ICU. A significant decrease in heart rate, respiratory rate, and FiO_2_ was found on the third day compared with the first day of admission. Based on the CONUT score, 53.8% of patients were at moderate risk of malnutrition, and 12.1% were at severe risk of malnutrition, which totals 65.9%.

Table 2 shows the results of the nutritional and biochemical parameters measured on the first and third days of admission and the evolution in that period in the ICU. A comparison test of means for related samples showed that prealbumin, transferrin, and cholesterol increase significantly on the third day of admission to the ICU, whereas total proteins, albumin, and TSI decrease significantly on the third day compared to the first day of admission to the ICU. Protein malnutrition (albumin < 3.5 g/dL) was evident in 61.4% (124 patients) of the patients on the day of admission, which was even more pronounced on the third day with 93.1% of the patients (188 patients). In 3 days, hypoalbuminemia increased in 30% of patients. Severe malnutrition (albumin < 2.1 g/dL) was observed in two patients on the first day (1% of patients). Prealbumin below 16 mg/dL was observed in 130 patients (64.4%) on the first day of admission.

Mortality at 28 days was 35.1% (71/202 patients). An association of nutritional and biochemical variables with mortality at 28 days was performed, as shown in Table 3. In this case, it was observed that albumin decreased significantly in those who died on the first and third days; that is, patients with the lowest albumin levels were more likely to die. We found that prealbumin on the third day of admission was also associated with mortality because its values were lower in the deceased than in the survivors.

**Figure 1:**
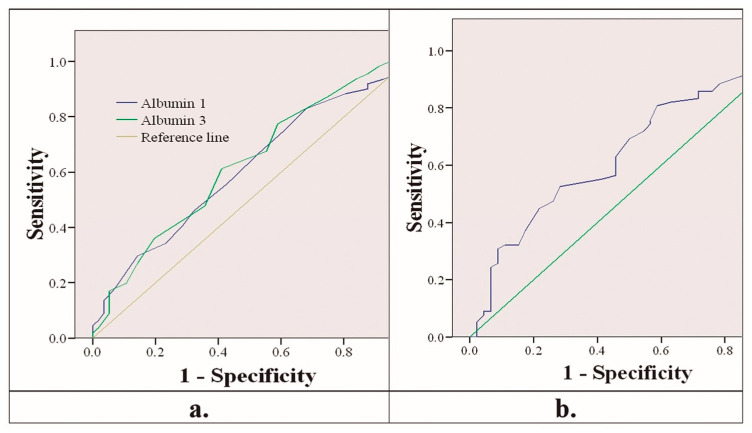
(**a**) ROC curve of albumin on the first and third days predicting mortality. (**b**) ROC curve of prealbumin on the third day predicting mortality.

Figure 1a shows the receiver operating characteristic curve (ROC curve) of albumin as a mortality predictor. The areas under the curve were 0.606 (95% CI 0.517–0.696) for albumin on the first day and 0.623 (95% CI 0.533–0.713) for albumin on the third day. In the same way, Figure 1b shows the ROC curve of prealbumin as a mortality predictor. The area under the curve was 0.637 (95% CI 0.538–0.737) for prealbumin on the third day.

**Figure 2:**
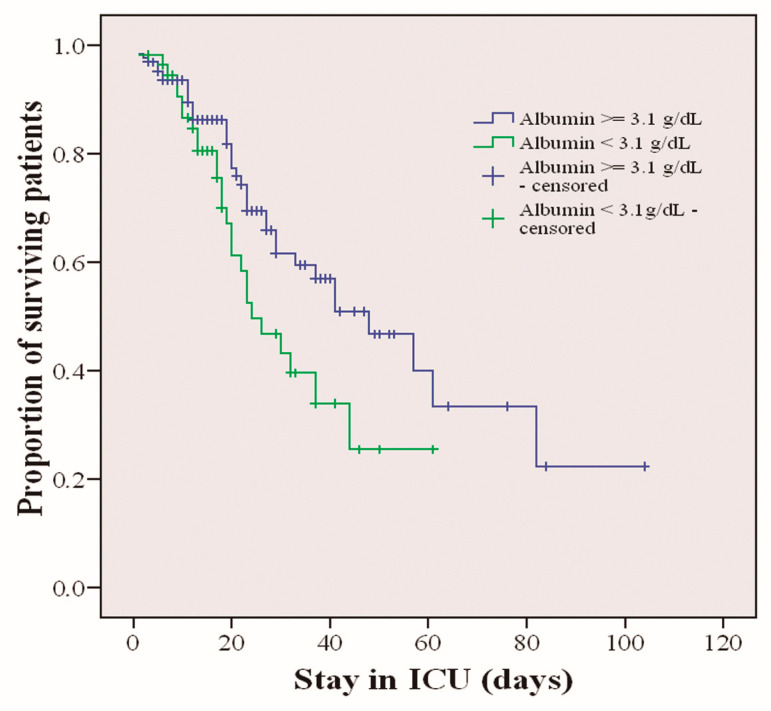
Survival of critically ill patients with COVID-19 stratified by risk of malnutrition based on albumin levels (*p* < 0.039).

The mean survival time was calculated for those patients who died. Survival was measured according to the greater or lesser risk of malnutrition. The risk of malnutrition was estimated with albumin values. A cut-off point was defined at 3.1 g/dL so that patients with albumin values higher than or equal to that value are defined as having a low risk of malnutrition, and patients with values lower than that value are defined as patients with a high risk of malnutrition. The median survival was 48.2 (95% CI 39.6–56.8). The association between the two groups with higher or lower risk of malnutrition and survival was calculated. A chi-square of 4.26 (*p* < 0.039) was obtained, so patients with a higher risk of malnutrition died at a higher percentage than those with a lower risk of malnutrition. Figure 2 shows this difference in survival.

Nutritional and biochemical parameters were related to some variables involved in the severity of the patient, such as SOFA, APACHE, CONUT, days of mechanical ventilation, and days of stay in the ICU. Table 4 shows the correlations found. Most are negative correlations; the lower the levels of nutritional parameters, the higher the severity of clinical variables.

Nutritional and biochemical parameters were also correlated with inflammation parameters such as fibrinogen, CRP, PCT, ferritin, and D-dimer. Table 5 shows the correlations found. Most are negative correlations; the lower the levels of nutritional parameters, the higher the levels of inflammatory markers.

## 4. Discussion

The main findings of the present study are the altered clinical parameters resulting from the critical state of patients with COVID-19, in addition to other nutritional biomarkers that responded more sensitively to the metabolic-nutritional changes suffered by critical patients. We found decreases in albumin levels over three days of admission to the ICU, whereas prealbumin levels have increased, probably due to the nutritional support performed in the ICU. A relationship between decreased albumin levels and mortality and a correlation between nutritional parameters and inflammatory parameters were found. This correlation was inverse, so the higher the inflammatory parameters, the lower the levels of nutritional parameters, except for triglycerides, where a direct correlation was found. Malnutrition in these patients and its association with severity have been previously reported in the literature [30].

Previous studies conducted on patients with COVID-19 admitted to the ICU have reported a prevalence of malnutrition consistent with our results, reaching 66.7% [31], but they used the GLIM criteria. The GLIM criteria [32] propose the evaluation of phenotypic criteria, including change in body weight, thinness (low body mass index), and reduced muscle mass, as well as criteria including poor nutritional intake and disease burden. GLIM criteria offer some advantages over ASPEN 2012 and ESPEN 2015 criteria. Although the above criteria are effective for diagnosing malnutrition, they are less useful in determining the severity of malnutrition. The GLIM criteria have high sensitivity, while the CONUT score can effectively predict the clinical outcome of malnutrition [33]. It is difficult to apply these criteria in clinical practice in the ICU because it is very difficult to obtain data on the weight of patients. For this reason, the CONUT scale is used more frequently to identify patients at risk of malnutrition in the ICU.As in our study, a relationship between CONUT score and mortality was demonstrated in other studies in patients with COVID-19 [34,35], in gastric cancer patients after curative resection [36], in patients hospitalized [37] for acute decompensated heart failure [38], in patients hospitalized for acute pulmonary embolism [39], in patients with infective endocarditis [40], in patients with heart failure [41], in patients with diffuse large cell lymphoma [42], in patients with traumatic brain injury [43]. It has also been seen that the increase in CONUT score is related to treatment failure for infections of the periprosthetic joint [44]. According to recent literature, it is recommended to evaluate the nutritional status of the patient infected with COVID-19 before admission [45] since itis potentially a reliable and independent prognostic indicator of mortality and length of hospitalization in COVID-19 patients [46]. A high percentage of patients admitted with COVID-19 are malnourished due to pre-existing conditions, including overweight and obesity. Since COVID-19 causes an acute decrease in intake both before admission and during their hospital stay, it is important to assess the risk of malnutrition as soon as possible after admission. It has been observed that low levels of markers of nutritional status are predictors of progression to respiratory failure and the need for mechanical ventilation [47]. Protein levels were significantly associated with adverse events [48]. In this same study, we can observe values very similar to ours for laboratory parameters of ICU patients with COVID-19; a mean albumin of 3.6 g/dL compared to 3.3 g/dL in our study, very similar transaminase levels, although slightly lower in our study, and an identical prealbumin level of 142 mg/dL [48]. Early enteral nutrition significantly reduces (*p* < 0.05) the risk of mortality among critically ill patients with COVID-19 [49], also seen in surgical patients [50].

Albumin is a parameter that provides information on the nutritional status of the patient but not on the disease progression. Albumin is a protein with a long half-life (20 days). Its levels are altered by non-nutritional factors such as infections, diffusion into the extracellular space, and dilution by infused fluids, which explains that albumin levels do not respond quickly to the nutrient replacement conducted in our study. Serum albumin levels decrease in the acute phase of infections and are associated with the intensity of the inflammatory response against them [51,52], as was found in our study. Decreased albumin levels are associated with an increased risk of acute respiratory failure and increased frequency of mechanical ventilation [53]. An association of albumin with mortality was also found in other studies; in COVID-19 patients [54], hip fracture patients [55,56], patients with infectious diseases [57], newborn patients [58], the lactate-to-albumin ratio in patients with acute myocardial infarction [59], the albumin-bilirubin score in patients with hypertrophic cardiomyopathy [60], a combined index of hemoglobin, albumin, lymphocyte, and platelet in patients with acute exacerbations of chronic obstructive pulmonary disease [61], and the albumin-to-prealbumin ratio in hemodialysis patients [62]. In malnourished patients, atrophy of the diaphragmatic muscle and loss of muscle mass can occur, in addition to a deterioration of the immune response, which leads to an increased risk of pneumonia. Decreased albumin may result in lower pulmonary oncotic pressure contributing to increased fluid extravasation [63,64,65], which, together with the higher vascular permeability underlying the respiratory insufficiency, would further worsen lung edema. A previous study reported serum albumin levels as an independent predictor of intra-abdominal infection after major hepatectomy [66]. Low serum albumin was also reported as a powerful predictor of cardiovascular adverse events in healthy subjects and patients with subclinical atherosclerosis [67]. An inverse association has also been found between albumin levels and depressive symptom scores in HIV-infected individuals [68]. It has been described in other articles that the clinical characteristics of patients with COVID-19 who have lower levels of serum albumin were related to a higher risk of death [69]. Low levels of albumin prevent it from performing its antioxidant role in the lungs, thus reducing its ability to fight oxidative stress and increasing lung injury [70]. Therefore, albumin levels can be used to assess the severity of infections in critically ill patients [71]. Albumin supplementation has not yet been shown to have beneficial effects in critically ill patients [72,73,74]. However, in a recent study with 350 pairs of septic patients with coronary heart disease, a relationship was observed between the administration of albumin and 28-day mortality from all causes; that is, survival was higher in the group of patients who received administered albumin than in the group that did not receive it [75].

Prealbumin has a short half-life, between 2 and 3 days, and is catabolized in the kidney. It is less affected by liver disease than other serum proteins. It has one of the highest ratios of essential to non-essential amino acids of any protein in the body, making it a distinctive marker for protein synthesis. Prealbumin is, therefore, the preferred marker of protein malnutrition. Due to its short half-life, prealbumin is a suitable marker to assess and monitor rapid changes, inflammatory burden, and nutritional status. Prealbumin can be used to monitor the patient’s nutritional intake during the hospital stay. In the present study, we found increases in prealbumin levels after three days. The association between mortality and prealbumin levels on the third day could indicate that those patients who have responded better to nutritional treatment, evidenced by the increase in prealbumin levels, have a better prognosis. Low serum prealbumin levels indicate the presence of acute inflammation and/or malnutrition, unlike other biomarkers such as CRP and PCT that predominantly reflect the inflammatory burden [76,77,78]. In consistency with our study, a previous study showed that prealbumin levels are inversely proportional to increased levels of CRP, characteristic of inflammation [79]. Increased serum prealbumin concentrations has been associated with favorable prognostic factors in hemodialysis patients [62].

In short, our study indicates that malnutrition in patients with COVID-19, evidenced by low albumin and prealbumin levels, is associated with clinical severity parameters and inflammatory parameters. Low serum albumin levels and other clinical and demographic characteristics may help identify patients at increased risk of severe disease and transfer to the ICU. Moreover, these levels could guide nutritional therapeutic strategies as an important element in ICU care [12]. Furthermore, monitoring the success of patients treated with adequate nutritional support using the prealbumin level as an indicator is possible.

## 5. Conclusions

The main finding of this study was the decrease in albumin and the increase in prealbumin three days after ICU admission of critical patients with COVID-19; in addition, a relationship was found between hypoalbuminemia, decreased serum prealbumin concentrations and CONUT, with high mortality in these patients. Measuring albumin upon admission of patients with COVID-19 would help to identify patients with a higher risk of malnutrition, and support measures could be adapted to the patients, improving the prognosis. The response to this enteral or parenteral nutrition can be monitored with prealbumin levels throughout the ICU stay. Therefore, a collaboration between nutritionists and intensivists is truly important for the detection, treatment, and follow-up of critically ill patients with COVID-19 at risk of malnutrition and, consequently, at risk of death.

## Figures and Tables

**Table 1:** Clinical characteristics of critical patients with COVID-19 on the first and third day of admission to the ICU.

*n* = 202	1st Day (Mean (SD))	3rd Day (Mean (SD))	*p*-Value
Age (years)	60.6 (13.6)		
ICU stay (days)	21.6 (16.6)		
MV (days) *	18.0 (10-29)		
SOFA score	6.52 (2.65)	7.00 (3.17)	0.158
APACHE II score	12.90 (5.32)		
CONUT score	5.91 (2.21)	6.12 (2.43)	0.467
MAP (mmHg)	91.1 (15.7)	89.4 (14.3)	0.566
HR (bpm)	80.2 (20.4)	68.7 (18.6)	0.001
BR (rpm)	26.8 (6.3)	22.0 (5.6)	0.001
FiO_2_ (%)	0.75 (0.18)	0.62 (0.16)	0.001
PaO_2_/FiO_2_	179 (88)	202 (70)	0.065

*p* < 0.05: Statistical significance. * It is expressed as the median (interquartile range) because it is a non-parametric variable. MV: mechanical ventilation. SOFA: sequential organ failure assessments. APACHE II: acute physiology and chronic health evaluation II. CONUT: controlling nutritional status. MAP: mean arterial pressure. HR: heart rate. BR: breathing rate. FiO_2_: fraction of inspired oxygen. PaO_2_/FiO_2_: partial pressure of oxygen/fraction of inspired oxygen.

**Table 2:** Nutritional and biochemical parameters of patients with COVID-19 on the first and third day of admission to the ICU.

*n* = 202	Reference Values	1st Day (Mean (SD)	3rd Day (Mean (SD)	*p*-Value
Biochemical variables				
Sodium (mEq/L)	136–146	138.5 (4.4)	140.6 (5.6)	0.001
Potassium (mEq/L)	3.5–5.1	4.03 (0.56)	4.01 (0.54)	0.679
Creatinine (mg/dL)	0.67–1.20	1.03 (0.73)	1.13 (0.93)	0.070
ALT (U/L) *	0–55	35.0 (23.0-59.5)	37.5 (25.0-72.7)	0.006
AST (U/L) *	5–40	37.0 (27.0-57.0)	31.0 (22.3-49.0)	0.005
GGT (U/L) *	1–55	73.0 (45-144)	107.0 (60-204.3)	0.001
LDH (U/L) *	0–248	525.0 (422.3-651.3)	445.5 (365.0-545.5)	0.001
Creatinekinase (U/L) *	0–190	88.0 (41.8-156.8)	62.5 (29.3-178.5)	0.311
Hematological variables				
Hemoglobin/dL	11.0–17.0	13.1 (2.0)	12.4 (2.4)	0.001
Hematocrit (%)	30.0–50.0	38.6 (6.3)	36.6 (6.0)	0.001
Leukocytes × 10^3^/µL	3.5–10.5	11.4 (5.5)	10.8 (5.1)	0.157
Lymphocytes (%)	20.00–44.00	7.60 (5.31)	9.85 (7.03)	0.001
Neutrophils (%)	42.00–77.00	86.3 (12.2)	82.4 (12.1)	0.001
Platelets × 10^3^/µL	3.5–10.5	250.5 (106.2)	284.0 (109.0)	0.001
INR	0.8–1.16	1.11 (0.15)	1.11 (0.19)	0.896
APTT (s)	26.0–37.0	29.5 (4.60)	29.4 (4.93)	0.823
Nutritional variables				
Glucose (mg/dL)	75–115	168.2 (70.1)	160.6 (66.7)	0.205
Total proteins (g/dL)	6.6–8.3	6.74 (4.14)	6.00 (1.03)	0.017
Albumin (g/dL)	3.5–5.2	3.28 (0.48)	3.0 (0.37)	0.001
Prealbumin (mg/dL)	16–42	14.2 (8.2)	28.3 (14.2)	0.001
Transferrin (mg/dL)	200–360	142.9 (32.2)	159.0 (46.9)	0.002
TSI (%)	17.1–30.6	43.4 (30.3)	36.2 (26.5)	0.018
Cholesterol (mg/dL)	140–200	145.8 (38.9)	179.7 (53.3)	0.001
Triglycerides (mg/dL)	89–150	285.9 (137.6)	319.2 (183.8)	0.154

*p* < 0.5: Statistical significance. * They are expressed as median (interquartile range) because they are non-parametric variables. ALT: alanine aminotransferase. AST: aspartateaminotransferase. GGT: glutamyltransferase. LDH: lactate dehydrogenase. INR: international normalized ratio. APTT: activated partial thromboplastin time. TSI: transferrin saturation index.

**Table 3:** Association of nutritional and biochemical parameters with 28-day mortality in patients with COVID-19.

*n* = 202	28-Day Mortality 1st Day			28-Day Mortality 3rd Day		
	Survivors (Mean ± SD)	Deceased (Mean ± SD)	*p*-Value	Survivors (Mean ± SD)	Deceased (Mean ± SD)	*p*-Value
Albumin (g/dL)	3.34 (0.49)	3.16 (0.46)	0.015	3.05 (0.37)	2.89 (0.37)	0.006
Prealbumin (mg/dL)	15.0 (8.3)	13.7 (8.0)	0.498	28.3 (13.2)	22.6 (11.5)	0.017
Transferrin (mg/dL)	146.2 (33.8)	145.4 (33.5)	0.913	159.7 (42.3)	147.1 (43.9)	0.117
TSI (%)	45.9 (27.3)	36.9 (32.4)	0.180	38.5 (25.1)	36.0 (25.5)	0.606
Cholesterol (mg/dL)	146.5 (38.3)	147.2 (40.4)	0.935	181.7 (50.7)	175.4 (60.5)	0.529
TG (mg/dL)	283.7 (155.8)	310.6 (160.9)	0.452	278.2 (135.4)	336.9 (228.8)	0.070
CONUT	5.7 (2.3)	6.1 (1.7)	0.475	5,9 (2.3)	6.7 (2.3)	0.043

*p* < 0.05: statistical significance. TSI: transferrin saturation index. TG: triglycerides. CONUT: controlling nutritional status scale.

**Table 4:** Correlations between nutritional and biochemical parameters and clinical variables.

	*n* = 202	SOFA	APACHE	CONUT	MV	ICU Stay
1st day	Albumin	−0.201 *	−0.162	−0.841 **	−0.089	0.024
	Prealbumin	−0.175	−0.253	−0.404 **	−0.173	−0.082
	Transferrin	−0.371 **	−0.200	−0.152	−0.023	−0.068
	TSI	−0.023	−0.094	−0.261 *	−0.259 *	−0.128
	Cholesterol	−0.268 *	−0.085	−0.305 **	0.033	0.101
	Triglycerides	0.225	0.031	−0.166	0.177	0.306 **
	CONUT	0.289 *	0.286		−0.063	−0.046
3rd day	Albumin	−0.111	−0.227	−0.829 **	−0.179 *	−0.120
	Prealbumin	−0.197	0.135	−0.364 **	0.139	−0.008
	Transferrin	−0.390 **	0.031	−0.394 **	−0.156	−0.071
	TSI	0.091	−0.149	0.124	−0.178 *	−0.111
	Cholesterol	−0.059	0.039	0.509 **	0.002	−0.034
	Triglycerides	0.285	0.029	−0.108	0.165	0.025
	CONUT	0.259	0.139		0.140	0.021

Statistical significance: * *p* < 0.05; ** *p* < 0.01. SOFA: Sequential Organ Failure Assessments. APACHE II: Acute Physiology and Chronic Health Evaluation II. CONUT: Controlling Nutritional Status scale. MV: Mechanic ventilation. ICU: Intensive Care Unit. TSI: Transferrin saturation index.

**Table 5:** Correlations between nutritional and biochemical parameters and inflammatory markers.

		Fibrinogen	D-Dimer	CRP	Ferritin
1st day	Albumin	0.080	−0.096	−0.070	−0.196 *
	Prealbumin	−0.205	0.213	−0.417 **	0.171
	Transferrin	−0.350 **	−0.043	−0.406 **	−0.508 **
	TSI	−0.030	0.090	−0.180	0.529 **
	Cholesterol	−0.006	0.085	−0.247 *	0.171
	Triglycerides	−0.109	0.236 *	−0.107	−0.027
	CONUT	0.066	−0.083	0.285 **	−0.157
3rd day	Albumin	−0.179 *	−0.075	−0.103	−0.133
	Prealbumin	−0.391 **	−0.106	−0.160	−0.016
	Transferrin	−0.338 **	−0.039	−0.155	−0.270 **
	TSI	−0.232 *	−0.070	−0.060	0.357 **
	Cholesterol	−0.032	−0.094	−0.145	−0.069
	Triglycerides	0.152	−0.021	−0.064	0.073
	CONUT	0.189 *	0.137	0.288 **	0.224 *

r = Correlation coefficient. Statistical significance: * *p* < 0.05; ** *p* < 0.01. TSI: Transferrin saturation index. CONUT: Controlling Nutritional Status.DD: D-dimer. CRP: C-reactive Protein.

## Data Availability

Data are available on request due to restrictions on privacy. The data presented in this study are available on request from the corresponding authors [Y.G.-M. and J.M.-L.].
